# Frequency and severity of malocclusions in patients with statutory health insurance in a German orthodontic practice in North Rhine Westphalia – a multi-part cross-sectional study over a 20-year period

**DOI:** 10.1007/s00784-023-05368-6

**Published:** 2023-11-29

**Authors:** Gero Stefan Michael Kinzinger, Jan Hourfar, Andrijana Maletic, Jörg Alexander Lisson

**Affiliations:** Department of Orthodontics, Saarland University, Homburg, Saar Germany

**Keywords:** Orthodontics, Malocclusion, Index system, KIG classification, Treatment need

## Abstract

**Objective:**

Since 2002, patients with statutory health insurance in Germany must undergo an assessment of orthodontic treatment need using the "Kieferorthopädische Indikationsguppen" (KIG; orthodontic indication groups) classification system. According to this system, tooth and jaw misalignment are divided into 11 subgroups and five grades. The objectives of this study were to determine the distribution of KIG classifications in patients with statutory insurance of a German orthodontic practice (North Rhine, Germany) and to analyze changes over a 20-year period.

**Materials and Methods:**

Since the introduction of the KIG index in 2002, 4940 statutorily insured patients over a 20-year period (2330 m, 2610 f, min 3.2, max 49.5 years, peak between 10 and 12 years) were classified at their first appointment. According to the valid guidelines of the statutory health insurance (GKV), the division was made into the highest possible KIG classification. Multiple entries were thus not made. In accordance with the operating cycles of the practice, the progression was divided into four 5-year periods.

**Results:**

Over a 20-year period, 24.98% of the patients were assigned to the classification "D". 86.52% of the patients were among the 6 most frequently ("D", "E", "K", "S", "P" and "M", > 10% each) and only 13.49% among the 5 least frequently recorded classifications ("U", "B", "T", "O" and "A", < 5% each).

**Conclusion:**

The distribution of the 6 most frequent and the 5 least frequent KIG classifications was constant over a 20-year-period. Among all possible tooth and jaw misalignment variants, the sagittal classifications "D" and "M" represent the most frequent malocclusions.

**Clinical relevance:**

The results and their comparison with historical data show that both frequency and severity of tooth and jaw misalignment with orthodontic treatment need appear identical for patients with statutory health insurance over a 20-year period.

## Introduction

Malocclusion related medical findings are, along with caries and gingivitis, among the most common oral health impairments in humans [1]. Their frequency shows global variations [2]. Population-wide representative data for children and adolescents from Germany are scarce. According to various historical cross-sectional studies from different German states [3–9] as well as the current DMS•6 [10–13], up to 97.5% of the children examined have malocclusions of the teeth and jaws in which orthodontic treatment may be indicated for medical reasons [10]. Depending on study design, objectively confirmed orthodontic treatment need exists in up to 51.7% [7] of the cases. When children and adolescents present themselves to orthodontists, their primary task is to detect any existing aberrations of a healthy masticatory system, tooth position and occlusal anomalies, and congenital deformities.

Since 2002, orthodontists in Germany must determine orthodontic treatment need during the initial examination of patients with statutory health insurance using a classification system called KIG [14]. In this, tooth and jaw misalignments were divided into 11 classifications. Each classification has five grades. Only patients with statutory health insurance presenting any classification with grade 3 and above are entitled to orthodontic treatment according to the social security code. The paragraph §29.1 of SGB V (»**S**ozial**g**esetz**b**uch« (SGB)»fünf« (V)) sets the legal framework and regulations for orthodontic treatment of statutorily insured patients in Germany. Comparable, predominantly degree-related indication group systems are also used in other European countries: the IOTN (Index of Orthodontic Treatment Need; [15]), the ICON (Index of Complexity, Outcome and Need; [16]), the SMBI (Swedish Medical Board Index; [17]) or the Swiss list of birth defects [18].

Until now, there has not yet been a long-term study on the frequency of individual indications, their distribution over long time periods, and possible regional differences in occurrence and severity among statutorily insured patients at the time of the initial orthodontic examination in Germany.

## Objective

The aim of the study was to investigate the frequency and expression of the existing 11 KIG classifications in an orthodontic practice from Viersen (North Rhine Westphalia, Germany) over a 20-year period. Without further subdivision according to age, gender and KIG grades, the aim was to determine if regional frequency and expression of medical findings requiring orthodontic treatment remain constant or not for patients with statutory health insurance, and to compare the data with existing epidemiologic data.

## Patients and Methods

The orthodontic practice where the data were collected was established by two orthodontists at the end of 2001. The practice was fully operational between 2002 and 2021, allowing a 20-year period to be scrutinized. The KIG classifications were done exclusively by two specialists for orthodontics during the 20-year practice period. All classifications were verified by the respective other orthodontist using the four-eye principle.

**Fig. 1:**
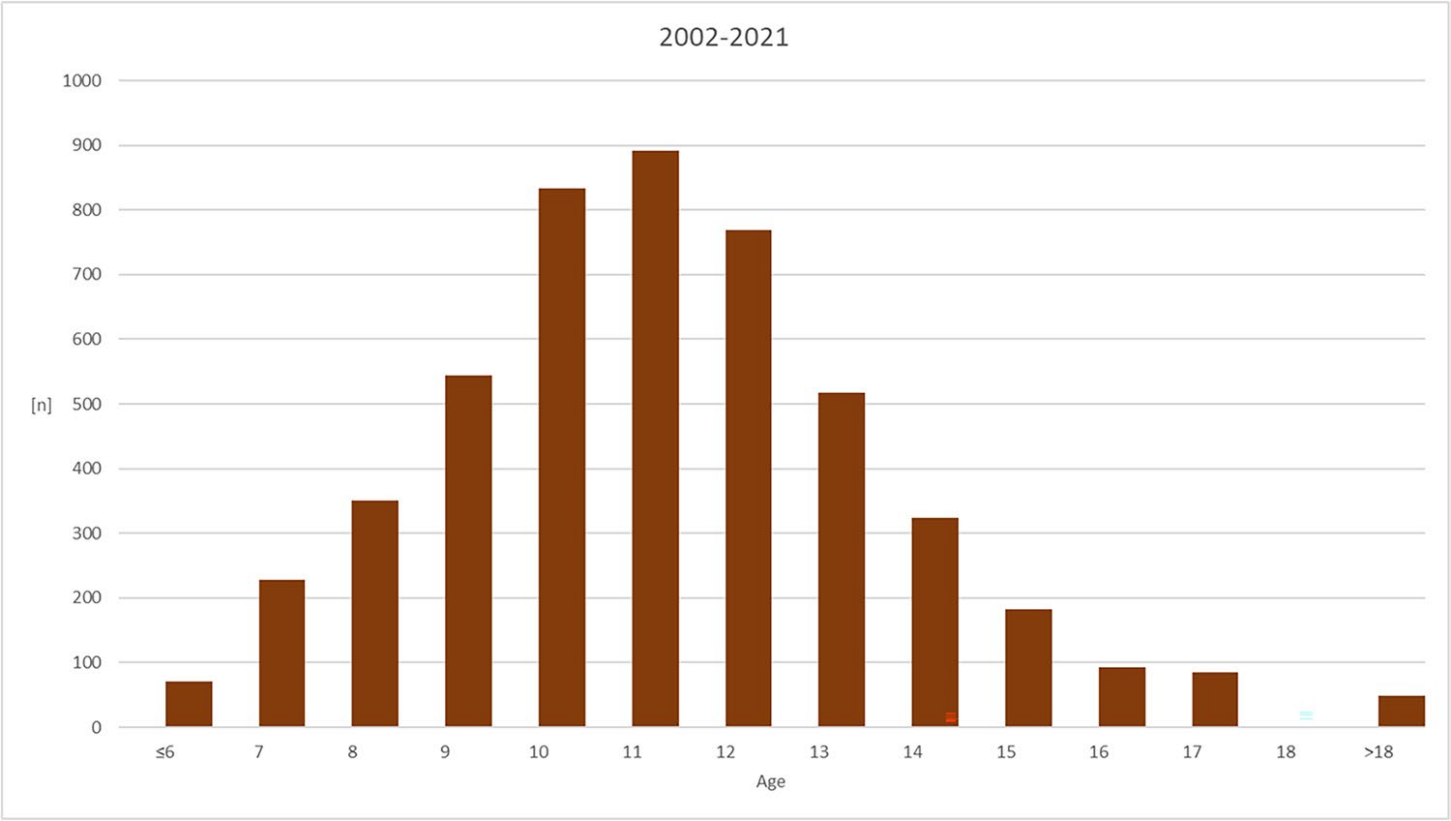
Age distribution of the 4940 statutorily insured patients at their first orthodontic consultation between 2002 and 2021

During a 20-year period after introduction of the KIG system, *n* = 4940 patients with statutory health insurance presented themselves and were subsequently assigned to the appropriate KIG classifications. *N* = 2330 patients were male, *n* = 2610 female (Table 1). Patient age ranged between min = 3.2 and max = 49.5 years, with an age peak between 10 and 12 years (Fig. 1, Table 2).

**Table 1:** Number of patients and gender distribution over a 20-year-period and divided into four five-year-periods between 2002–2021

Period	female		male		total	
	[n]	[%]	[n]	[%]	[n]	[%]
2002–2006	627	54,29	528	45,71	1155	100
2007–2011	782	52,24	715	47,76	1497	100
2012–2016	706	52,96	627	47,04	1333	100
2017–2021	495	51,83	460	48,17	955	100
2002–2021	2610	52,83	2330	47,17	4940	100

**Table 2:** Age distribution over a 20-year-period and divided into four five-year-periods between 2002–2021

	Patients age, all patients [yrs]			Age Category [yrs]													
Period	Mean	Min	Max	≤ 6	7	8	9	10	11	12	13	14	15	16	17	18	> 18
2002–2006	11.16	3.21	39.56	21	77	103	168	235	206	156	82	46	24	10	16	0	11
2007–2011	11.53	4.45	42.33	21	64	111	168	252	313	246	146	87	44	24	13	1	7
2012–2016	12.19	5.80	49.51	15	48	79	105	195	220	222	187	114	72	33	25	1	17
2017–2021	12.01	3.56	44.15	14	39	58	103	151	152	145	103	77	43	26	31	0	13
2002–2021	11.71	3.21	49.51	71	228	351	544	833	891	769	518	324	183	93	85	2	48

**Fig. 2:**
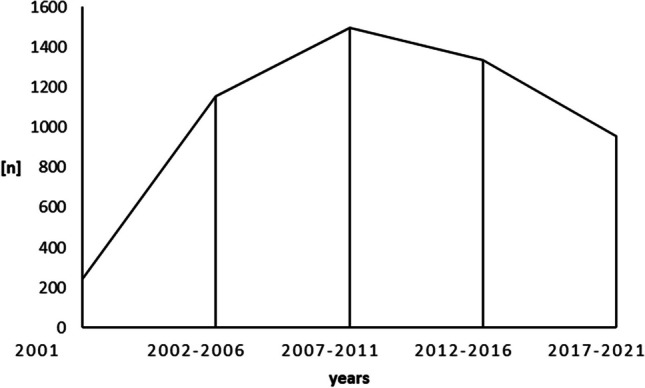
Working periods of the practice with subdivision into 4 × 5 years. *N* = frequency of KIG assessments for patients with statutory health insurance

Apart from the foundation (2001) and handover (2022) periods, the progression is divided into four 5-year-periods (Fig. 2, Table 1) according to the operating cycles of the practice:

Period I:2002–2006: 1155 patients; practice growth and consolidation period.

Period II:2007–2011: 1497 patients; practice working period.

Period III:2012–2016: 1333 patients; practice working period.

Period IV:2017–2021: 955 patients; practice working and shutdown period.

## Classification of orthodontic treatment need using the German index»Kieferorthopädische Indikationsgruppen (KIG)«

Within the KIG index system, tooth position and jaw anomalies are subdivided into 11 classifications of decreasing complexity. The classifications are additionally subdivided into five grades of severity. Table 3 shows the KIG classification ranking, with A being the highest and P the lowest possible classification. Only classifications with grades 3–5 are eligible for treatment within the framework of the statutory health system.

**Table 3:** KIG classification ranking, with A being the highest and P the lowest possible classification. The original German definition is in the second column

KIG classification	Classification description	German description
A	Craniofacial Anomalies (Cleft palate and syndromes)	Kraniofaziale **A**nomalien
U	Missing teeth (Agenesis or loss)	Zahn**u**nterzahl
S	Disturbance in tooth eruption (Impaction, Displacement)	Durchbruchs**s**törungen
D	Sagittal discrepancy increased overjet	**D**istale Stufe
M	Sagittal discrepancy negative overjet	**M**esiale Stufe
O	Vertical discrepancy open bite (habitually open / skeletally open)	**O**ffener Biss
T	Vertical discrepancy deep bite (with / without mucosal contact; with traumatic mucosal impingement)	**T**iefer Biss
B	Transverse discrepancy (Scissors bite)	**B**ukkal-, Lingualokklusion
K	Transverse discrepancy crossbite (Buccolingually cusp-to-cusp relation, Bilateral crossbite, Unilateral crossbite)	**K**reuzbiss
E	Contact point displacement	**E**ngstand
P	Space deficiency	**P**latzmangel

The diagnoses were solely recorded through clinical inspection, as required by legislation. The extent and direction of sagittal and vertical overjet, anterior crowding and space deficits were measured intraorally. All measurements were performed using an orthodontic caliper»Münchner Modell®« (Dentaurum, Ispringen, Germany) with a precision of 0.25 mm. The assessment of occlusion regarding frontal and lateral crossbites was performed visually. Only if justified by clinical reasons, panoramic x-rays were taken to diagnose possible aplasia, retention, or displacement of permanent teeth. The diagnosis was made regardless of patient age at initial presentation. The classification of the registered diagnoses was performed according to the valid GKV guidelines [5]. Multiple entries were thus not made, but the allocation was made according to the highest possible KIG classification and grade.

## Statistics

The anonymized data were collected in a structured manner using spreadsheet software (Microsoft Excel®, Microsoft Corp., Redmond, Washington, USA), interpreted descriptively and presented.

## Results

### Entire 20-year-period (Fig. 3, Table 4)

**Fig. 3:**
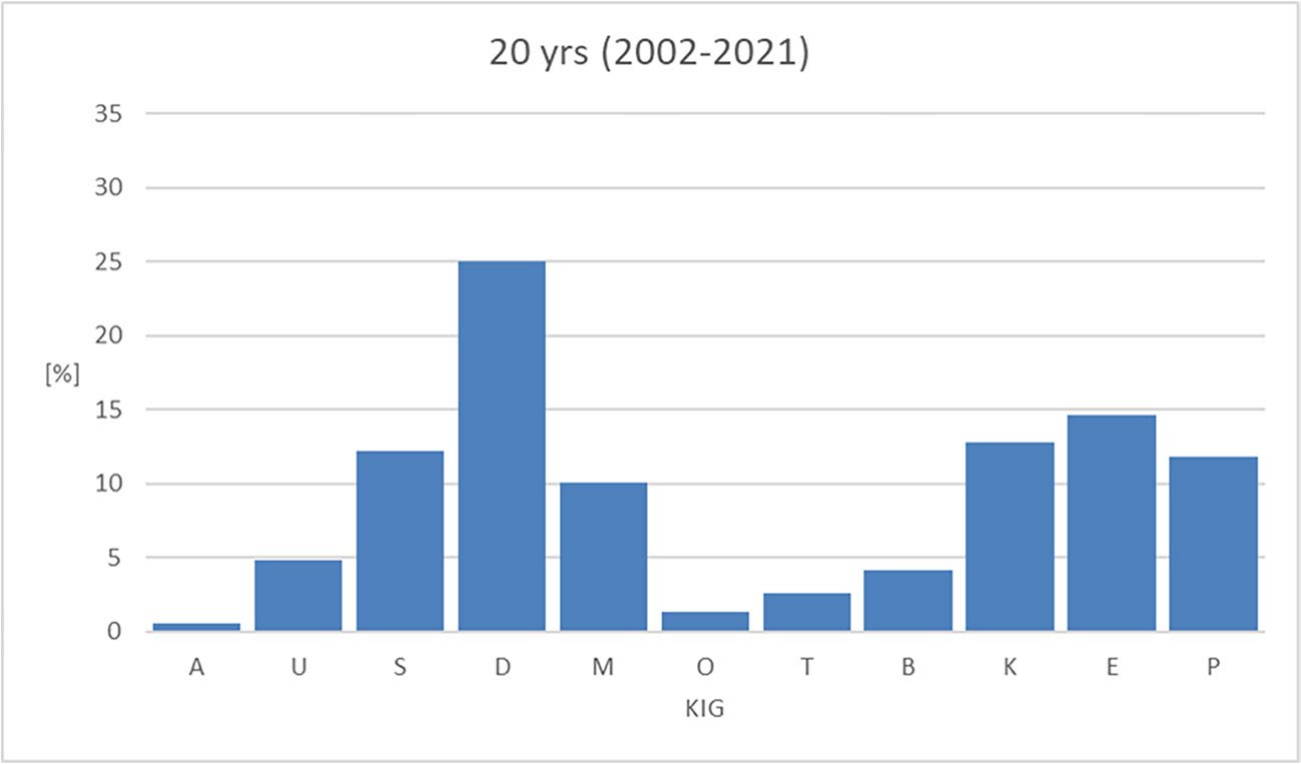
Percentage of the 11 KIG classifications in patients with statutory health insurance between 2002–2021

**Table 4:** Distribution of 11 possible KIG classifications over a 20-year-period and divided into four five-year-periods between 2002–2021

KIG Classification	5 yrs total 2002–2006 [n]	5 yrs total 2007–2011 [n]	5 yrs total 2012–2016 [n]	5 yrs total 2017–2021 [n]	20 yrs total 2002–2021 [n]	20 yrs total 2002–2021 [%]
A	13	4	5	4	26	0.53
U	78	59	55	46	238	4.82
S	135	244	134	88	601	12.17
D	221	353	389	271	1234	24.98
M	139	149	122	90	500	10.12
O	20	21	20	8	69	1.40
T	44	25	37	21	127	2.57
B	36	72	56	42	206	4.17
K	157	194	156	127	634	12.83
E	176	165	224	157	722	14.62
P	136	211	135	101	583	11.80
Total	1155	1497	1333	955	4940	100.00

Over the entire 20-year-period, 24.98% of the patients (1234 out of 4940) were allocated to KIG classification "D" (increased overjet). Proportionally more than 10% were distributed among the KIG classifications "E" (722 patients, 14.62%), "K" (634 patients, 12.83%), "S" (601 patients, 12.17%), "P" (583 patients, 11.80%), and "M" (500 patients, 10.12%). Less than 5% was accounted for by KIG classifications "U" (238 patients, 4.82%), "B" (206 patients, 4.17%), "T" (127 patients, 2.57%), and "O" (69 patients, 1.40%), and less than 1% was accounted for "A" (26 patients, 0.53%). The 3 most frequent KIG classifications ("D", "E" and "K") accounted for 52.43% of the patients.

Considering all 11 KIG classifications, 86.52% were distributed among the 6 most frequent ("D", "E", "K", "S", "P" and "M", each more than 10%) and only 13.49% among the 5 rarest findings ("U", "B", "T", "O" and "A", each less than 5%).

### Observation according to 5-year periods (Figs. 4a-d and 5, Tables 5, 6, 7, 8)

**Fig. 4:**
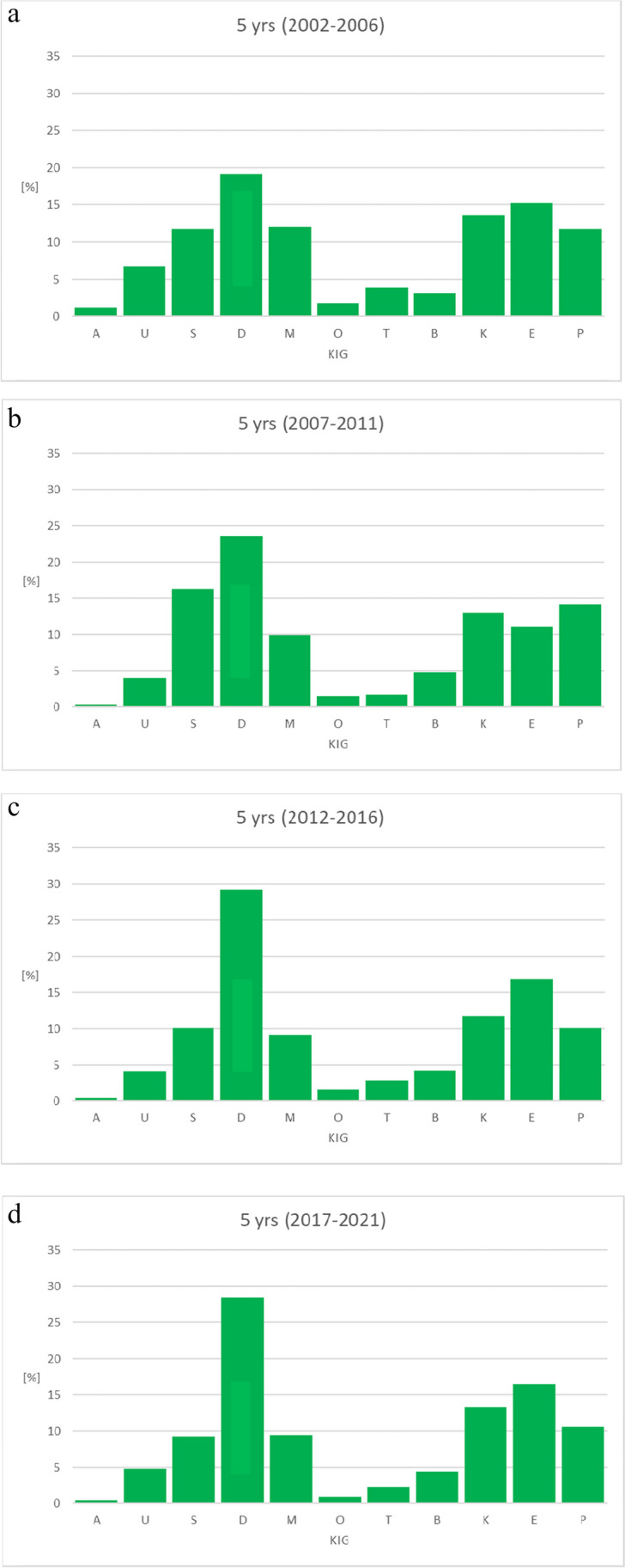
a Percentage of the 11 KIG classifications in patients with statutory health insurance in practice period I between 2002–2006. b Percentage of the 11 KIG classifications in patients with statutory health insurance in practice period II between 2007–2011. c Percentage of the 11 KIG classifications in patients with statutory health insurance in practice period III between 2012–2016. d Percentage of the 11 KIG classifications in patients with statutory health insurance in practice period IV between 2017–2021

**Fig. 5:**
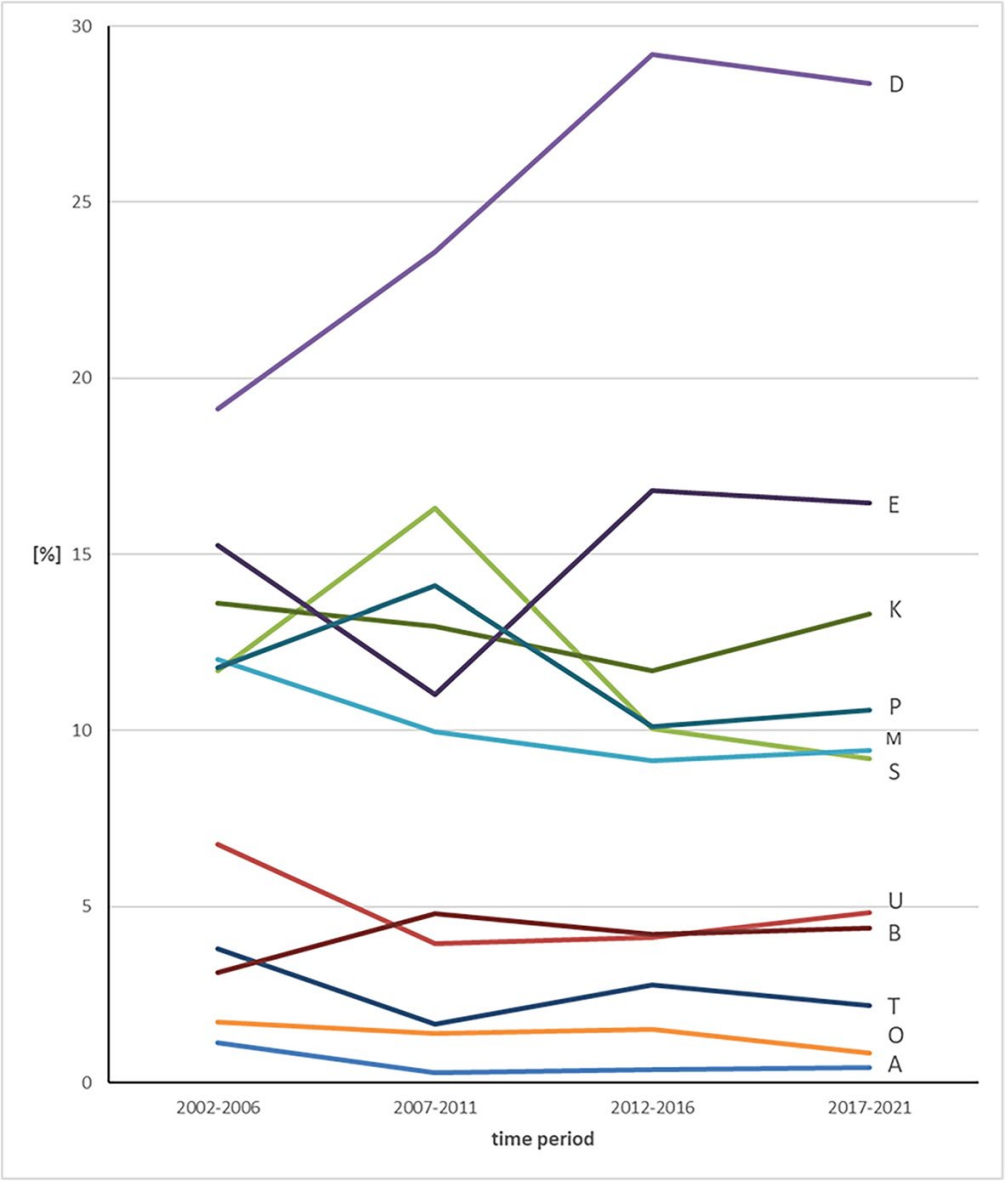
Percentage of the 11 KIG classifications in patients with statutory health insurance during four five-year-periods

**Table 5:** Distribution of 11 possible KIG classifications during practice period I between 2002–2006

KIG classification	2002 [n]	2002 [%]	2003 [n]	2003 [%]	2004 [n]	2004 [%]	2005 [n]	2005 [%]	2006 [n]	2006 [%]	5 yrs total [n]	5 yrs total [%]
A	3	2,08	4	1,84	3	1,49	2	0,74	1	0,31	13	1,13
U	11	7,64	22	10,14	11	5,47	16	5,90	18	5,59	78	6,75
S	13	9,03	34	15,67	29	14,43	30	11,07	29	9,01	135	11,69
D	32	22,22	29	13,36	31	15,42	52	19,19	77	23,91	221	19,13
M	19	13,19	26	11,98	26	12,94	31	11,44	37	11,49	139	12,03
O	2	1,39	6	2,76	4	1,99	4	1,48	4	1,24	20	1,73
T	11	7,64	23	10,60	2	1,00	6	2,21	2	0,62	44	3,81
B	3	2,08	4	1,84	5	2,49	14	5,17	10	3,11	36	3,12
K	19	13,19	29	13,36	35	17,41	33	12,18	41	12,73	157	13,59
E	17	11,81	25	11,52	34	16,92	40	14,76	60	18,63	176	15,24
P	14	9,72	15	6,91	21	10,45	43	15,87	43	13,35	136	11,77
Total	144	100,00	217	100,00	201	100,00	271	100,00	322	100,00	1155	100,00

**Table 6:** Distribution of 11 possible KIG classifications during practice period II between 2007–2011

KIG classification	2007 [n]	2007 [%]	2008 [n]	2008 [%]	2009 [n]	2009 [%]	2010 [n]	2010 [%]	2011 [n]	2011 [%]	5 yrs total [n]	5 yrs total [%]
A	2	0,50	1	0,38	1	0,33	0	0	0	0,00	4	0,27
U	16	4,02	15	5,75	12	3,93	10	3,89	6	2,17	59	3,94
S	66	16,58	35	13,41	63	20,66	41	15,95	39	14,13	244	16,30
D	86	21,61	77	29,50	68	22,30	53	20,62	69	25,00	353	23,58
M	47	11,81	30	11,49	25	8,20	23	8,95	24	8,70	149	9,95
O	8	2,01	2	0,77	4	1,31	5	1,95	2	0,72	21	1,40
T	1	0,25	5	1,92	3	0,98	8	3,11	8	2,90	25	1,67
B	20	5,03	7	2,68	15	4,92	22	8,56	8	2,90	72	4,81
K	52	13,07	29	11,11	42	13,77	30	11,67	41	14,86	194	12,96
E	56	14,07	18	6,90	28	9,18	28	10,89	35	12,68	165	11,02
P	44	11,06	42	16,09	44	14,43	37	14,40	44	15,94	211	14,09
Total	398	100,00	261	100,00	305	100,00	257	100,00	276	100,00	1497	100,00

**Table 7:** Distribution of 11 possible KIG classifications during practice period III between 2012–2016

KIG classification	2012 [n]	2012 [%]	2013 [n]	2013 [%]	2014 [n]	2014 [%]	2015 [n]	2015 [%]	2016 [n]	2016 [%]	5 yrs total [n]	5 yrs total [%]
A	3	0,98	0	0,00	0	0,00	2	0,82	0	0,00	5	0,38
U	14	4,56	13	4,92	5	2,01	9	3,70	14	5,19	55	4,13
S	32	10,42	29	10,98	34	13,65	16	6,58	23	8,52	134	10,05
D	106	34,53	77	29,17	68	27,31	65	26,75	73	27,04	389	29,18
M	23	7,49	21	7,95	32	12,85	24	9,88	22	8,15	122	9,15
O	4	1,30	2	0,76	3	1,20	5	2,06	6	2,22	20	1,50
T	12	3,91	1	0,38	2	0,80	9	3,70	13	4,81	37	2,78
B	12	3,91	15	5,68	9	3,61	9	3,70	11	4,07	56	4,20
K	27	8,79	27	10,23	37	14,86	32	13,17	33	12,22	156	11,70
E	47	15,31	40	15,15	40	16,06	48	19,75	49	18,15	224	16,80
P	27	8,79	39	14,77	19	7,63	24	9,88	26	9,63	135	10,13
Gesamt	307	100,00	264	100,00	249	100,00	243	100,00	270	100,00	1333	100,00

**Table 8:** Distribution of 11 possible KIG classifications during practice period IV between 2017–2021

KIG classification	2017 [n]	2017 [%]	2018 [n]	2018 [%]	2019 [n]	2019 [%]	2020 [n]	2020 [%]	2021 [n]	2021 [%]	5 yrs total [n]	5 yrs total [%]
A	1	0,43	1	0,61	1	0,51	0	0,00	1	0,56	4	0,42
U	10	4,31	5	3,07	9	4,59	8	4,30	14	7,87	46	4,82
S	14	6,03	10	6,13	20	10,20	21	11,29	23	12,92	88	9,21
D	73	31,47	37	22,70	53	27,04	61	32,80	47	26,40	271	28,38
M	25	10,78	13	7,98	17	8,67	20	10,75	15	8,43	90	9,42
O	5	2,16	1	0,61	2	1,02	0	0,00	0	0,00	8	0,84
T	8	3,45	5	3,07	2	1,02	3	1,61	3	1,69	21	2,20
B	10	4,31	6	3,68	12	6,12	8	4,30	6	3,37	42	4,40
K	32	13,79	30	18,40	28	14,29	20	10,75	17	9,55	127	13,30
E	27	11,64	40	24,54	36	18,37	28	15,05	26	14,61	157	16,44
P	27	11,64	15	9,20	16	8,16	17	9,14	26	14,61	101	10,58
Gesamt	232	100,00	163	100,00	196	100,00	186	100,00	178	100,00	955	100,00

- "D" was the most frequent classification in all evaluated periods; in period I initially below 20%, increasing up to almost 30% in periods III and IV.
- "S" was significantly higher, "P" was slightly increased, "E" was significantly lower during period II.
- The three most frequent classifications D + E + K had an increase of about 10% in periods III and IV compared to periods I and II (I 47.96%, II 47.56%, III 57.68%, IV 58.12%).
- The distribution between the 6 most frequent and the 5 least frequent KIG classifications remained constant during all observed periods.

The analysis of the 6 most frequent KIG classifications reveals the following:

- D remained stable at a high level over periods III and IV.
- E decreased first, then increased again and remained constant around 16%.
- K occurred relatively constant between 11.70% and 13.59%.
- P occurred relatively constant between 10.13% and 14.09%.
- M occurred relatively constant between 9.15% and 12.03%.
- S increased from 11.69% to 16.30%, then decreased down to 9.21%

The analysis of the 5 least frequent KIG classifications reveals the following:

- B always remained below 5%
- U was first above, then constantly below 5%
- T was always below 5%
- O was always the second rarest KIG classification and below 2%
- A was always the rarest KIG classification, initially just above 1%, then well below 1%

### Combination of classifications in the respective spatial planes

- Among sagittal classifications, "D" is always the most frequent, with > 20% during 3 periods and nearly 30% during the last two periods. "M" occurs constantly around 10%.
- The vertical classifications "O" and "T" always occur below 5%.
- In the transverse classifications, "B" is always below 5%, while "K" occurs constantly above 10%.

## Discussion

Longitudinal studies on frequency and severity of dental malocclusions are not available from orthodontic practices in Germany. Rather, cross-sectional studies on selected groups of patients from age groups with a limited time span have been conducted in various regions of Germany over the past 25 years to determine the frequency of anomalies and the associated need for orthodontic treatment.

Bäßler-Zeltmann et al. [3] published a study of 1020 German schoolchildren between 8.5 and 9.5 years in 1998. The frequency of malocclusions and the need for orthodontic treatment were investigated. The criterion was the Swedish Medical Authorities' five-degree scale, which was used in a clinical-only examination. Multiple responses (dentition anomalies, space anomalies, and occlusion anomalies) were possible. Crowding in one or more segments was noted in 53.4%, and midline shift in 26% of patients. Tooth rotations ≥ 30° were found in 19%, a pronounced deep bite in 7.3%, a crossbite of the anterior teeth in 5.1%, and a frontal open bite in 3.5% of the children. 56% of all children had neutral occlusion. Orthodontic treatment was necessary in 32% and desirable in another 32% according to the applied scale.

In 2000, Schopf [4] investigated orthodontically relevant findings in 2326 pupils aged 6 to 7 years at the onset of the early mixed dentition. Six criteria were investigated: vertical overjet, sagittal overjet, occlusion, crossbite, supporting zones, anterior crowding. Multiple responses were possible. The examination results were divided into 3 classifications. Accordingly, 14.7% of the children had no pathological findings. 77.2% had findings of low to high severity with no indication for early onset treatment. In 8.04%, the initiation of appliance-based orthodontic measures was considered immediately necessary.

After the introduction of the KIG classification, Glasl et al. [5] examined 1251 pupils aged 9 to 11 years in 2004 as part of a follow-up study [4]. 12.1% of the subjects were already receiving treatment at the time of the study, with more than half still having a KIG grade ≥ 3. Most subjects were recruited from participants in Schopf's study [4]. The 50% decrease in the number of subjects was due to private reasons of the participants. Findings were also collected clinically only, i.e., in accordance with current legislation, without radiographic records or dental casts. Multiple recording of different existing KIG classifications of each study participant was possible. Among the tooth position and jaw anomalies detected and requiring treatment, the lateral crossbite dominated with 9.2% and the enlarged overjet (> 6 mm) with 8.7%. Among all registered anomalies with KIG grades ≥ 3, the most common categories out of 19 possible variants were KIG D grade 4 (D4: overjet > 6 mm) with 17.4%, KIG K grade 4 (K4: unilateral crossbite) with 15.3%, and KIG M grade 4 (M4: reverse overjet ≤ -3 mm) with 14.9%. 10.6% had pronounced malocclusions (KIG grade 3), 29.4% pronounced malocclusions (KIG grade 4), and 1.4% extremely pronounced malocclusions (KIG grade 5). A treatment indication in the sense of the statutory health insurance (KIG ≥ 3) existed in 41.4% and was thus significantly higher than the 8.0% suggested by Schopf [4].

In 2004, Assimakopoulou [6] investigated the need for orthodontic treatment of 526 9- to 10-year-old elementary school children from Münster / Westphalia using dental casts. 266 subjects were males (51%) and 260 (49%) females. The dental casts were evaluated through electronic model analyses and additional manual measurements. 46% of the study subjects needed treatment according to the KIG classification. 28% of the participants had not yet received treatment at the time of the examination, and 18% were already undergoing orthodontic treatment. Among the untreated, the following findings with KIG grades ≥ 3 were represented: "D" 7%, "O" 3%, "T" 22%, "E" 14%, "P" 5%. Among those already treated, the following findings KIG grades ≥ 3 were represented "D" 8%, "M" 1%, "O" 1%, "T" 23%, "E" 14%, "P" 7%. The classifications "A", "U" and "S" were not analyzed.

In 2004, Tausche et al. [7] published a study on the prevalence of malocclusions in the early mixed dentition in 1975 patients aged between 6 and 8 years using the IOTN. The study was part of a study already planned in 1997 and 1998 with a total of 8768 school children from Dresden. The classification was based on seven criteria (vertical overjet > 3 mm, sagittal overjet > 3 mm, crossbite, open bite > 1 mm, crowding maxilla, crowding mandibula, Class III). Multiple responses were also possible in this study. For the interpretation of the study results, a subdivision into five grades (none, little, borderline need, treatment need, very great treatment need) was performed. The results show that deep overbite (> 3.5 mm) with 46.2% and overjet (> 3 mm) with 37.5% were the most common malocclusions, followed by anterior open bite (17.7%), crossbite (8.2%) and reverse overbite (3.2%). Grades 4 and 5 combined with treatment needs reached 26.2%, grade 3, 4 and 5 together 51.7%.

In 2007, Grabowski et al. [8] compared the distribution of malocclusions in 766 children in primary dentition (mean age 4.5 years) with 2275 children with mixed dentition (mean age 8.3 years). Sagittal, transverse and vertical dental anomalies and additional occlusal findings were recorded. Multiple responses were possible. Symmetric distal occlusion was present in 25.8% of children in the primary dentition and in 31.4% of children in the early mixed dentition. Enlarged overjet of > 6 mm had only 3.2% and 4.2% of the children, respectively. Reduced overjet ≤ 0 mm occurred in 3.3% and 5.3%, respectively. From the primary to the mixed dentition, the occurrence of lateral crossbites also increased significantly from 7.2% to 12.0%.

Lux et al. [9] examined 494 schoolchildren in the south-west of Germany with an average age of 9 years (8.6 years to 9.6 years) for the prevalence of occlusal anomalies. Overjet, overbite, sagittal molar relationships, crossbites, scissor bites and midline displacements were evaluated. An increased overjet was generally found more frequently than a reduced or reversed overjet, with a greatly increased overjet > 6 mm affecting approximately 5–10% of the children. There was considerable variation in overbite between -1 mm and 9 mm. Patients with Class II malocclusion had significantly increased overbite compared to individuals with Class I malocclusion. Traumatic contact of the gingiva affected one in 14 children. Distal occlusion greater than half a premolar width or more affected more than 20% of children. In contrast, only 3% of children aged 9 years had a mesial occlusion of at least half a premolar width.

As part of the Sixth German Oral Health Study (DMS•6), a representative epidemiological survey of the prevalence of tooth and jaw misalignment was conducted for the first time population-wide in Germany in the group of 8- to 9-year-olds. The results of this study were published in 2023 [10–13]. The primary objective of this study was to identify tooth and jaw misalignment in 8- and 9-year-old children. The secondary objective was to determine the possible need for orthodontic care. Findings were obtained and evaluated from 705 study participants (51.4% male, 48.6% female). The proportion of 8-year-olds was 49.4%, that of 9-year-olds 50.6%. The KIG classifications "U" and "S were not recorded because no radiographs were taken. Multiple responses were possible for the remaining 9 KIG classifications. The part of study participants for whom orthodontic treatment is indicated according to current statutory health insurance guidelines (KIG grades 3—5) was 40.4%. Of these, 10.0% had pronounced malocclusions (KIG grade 3), 25.5% had very pronounced malocclusions (KIG grade 4), and 5.0% had extremely pronounced malocclusions (KIG grade 5). Individually, increased overjet occurred with 88.9%. Crowding was also widespread, with at least 60.9%, as was space deficiency, with a share of 30.3%. All other indication groups had a share of less than 10% each.

A comparison of the present results with previously conducted studies is only possible to a limited extent, since the studies were based on different parameters—different index, multiple responses, partial omission of individual findings—and the study population was different. In the single-subject cross-sectional studies, the age of the subjects was clearly defined, but the subject population was not preselected. The patients in the present multi-part cross-sectional study were predominantly referred to orthodontic care by external dentists over a 20-year-period and are therefore only representative to a limited extent. In contrast to the other studies, there were no study-related limitations in the diagnosis, so that the criteria missing teeth and tooth retention could also be assessed.

However, the results of the present unicentric study document a consistently high need for initial orthodontic consultation and treatment among statutorily insured patients with a largely constant distribution of anomalies over the entire 20-year observation period. This indirectly confirms the hypothesis established by the comparison of the one-stage unicentric study by Glasl et al. [5], and the current one-stage multicenter DMS•6 study that the need for orthodontic treatment in Germany has remained largely constant over the years [10].

### Possible limitations of the study

In the present study, the KIG findings were collected by different orthodontists. According to Gesch et al. [19], there are considerable examiner differences in the classification of subjects into the correct orthodontic indication groups. Different elicitation methods (clinic/model) in the reporting of malocclusion symptoms by several examiners as well as examiners inexperienced in orthodontics may have an unfavorable influence on examiner agreement. For this reason, all KIG classifications and treatment plans were reviewed and validated by another orthodontist applying the four-eye-principle throughout. In the case of grade discrepancies that could not be eliminated (KIG ≤ 2 vs. KIG ≥ 3), the classifications were made based on a dental cast and, if necessary, an additional x-ray.

## Conclusion

The present study was the first to evaluate, without study-related limitations, the frequency and severity of tooth and jaw misalignment in patients at their first appointment over a 20-year period in accordance with current legal guidelines in an orthodontic specialty dental practice. "D" was the most frequently detected classification in all observed 5-year intervals. The 3 most frequent classifications – "D", "E" and "K" – showed an increase of approximately 10% over the second 10 years compared with the first 10 years. The distribution of the 6 most common and the 5 least common KIG classifications was constant through the entire 20-year period. When subdivided by tooth position and jaw anomalies, the sagittal deviations "D" and "M" represented the most common malocclusions.

## Data Availability

Not applicable.

## References

[1] Dhar V, Jain A, Van Dyke TE, Kohli A (2007). Prevalence of gingival diseases, malocclusion and fluorosis in school-going children of rural areas in Udaipur district. J Indian Soc Pedod Prev Dent.

[2] Alhammadi MS, Halboub E, Fayed MS, Labib A, El-Saaidi C (2018). Global distribution of malocclusion traits: a systematic review. Dental Press J Orthod.

[3] Bäßler-Zeltmann S, Kretschmer I, Göz G (1998). Malocclusion and the need for orthodontic treatment in 9-year-old children. J Orofac Orthop/Fortschr Kieferorthop.

[4] Schopf P (2003). Indication for and frequency of early orthodontic therapy or interceptive measures. J Orofac Orthop/Fortschr Kieferorthop.

[5] Glasl B, Ludwig B, Schopf P (2006). Prevalence and development of KIG-relevant symptoms in primary school students from Frankfurt am Main. J Orofac Orthop.

[6] Assimakopoulou T (2004) Evaluierung der Prävalenzrate bei 9 bis 10-jährigen Probanden nach den Kieferorthopädischen Indikationsgruppen (KIG). Doctoral Thesis. Westfälische Wilhelms-Universität, Münster

[7] Tausche E, Luck O, Harzer W (2004). Prevalence of malocclusions in the early mixed dentition and orthodontic treatment need. Eur J Orthod.

[8] Grabowski R, Stahl F, Gaebel M, Kundt G (2007). Relationship between occlusal findings and orofacial myofunctional status in primary and mixed dentition. Part I: Prevalence of malocclusions. J Orofac Orthop.

[9] Lux CJ, Ducker B, Pritsch M, Komposch G, Niekusch U (2009). Occlusal status and prevalence of occlusal malocclusion traits among 9-year-old schoolchildren. Eur J Orthod.

[10] Jordan AR, Kuhr K, Frenzel Baudisch N, Kirschneck C (2023). Prevalence of malocclusions in 8- and 9-year-old children in Germany-results of the sixth german oral health study (DMS 6). J Orofac Orthop.

[11] Jordan AR, Kuhr K, Ohm C, Frenzel Baudisch N (2023). Methodology of the sixth german oral health study (DMS 6) to survey tooth and jaw misalignment. J Orofac Orthop.

[12] Bekes K, Kuhr K, Ohm C, Frenzel Baudisch N, Jordan AR (2023). Does orthodontic treatment need have an impact on oral health-related quality of life?. J Orofac Orthop.

[13] Kirschneck C, Kuhr K, Ohm C, Frenzel Baudisch N, Jordan AR (2023). Comparison of orthodontic treatment need and malocclusion prevalence according to KIG, ICON, and mIOTN in German 8- to 9-year-old children of the Sixth German Oral Health Study (DMS 6). J Orofac Orthop.

[14] Schopf P (2001). Die kieferorthopädischen Indikationsgruppen.

[15] Brook PH, Shaw WC (1989). The development of an index of orthodontic treatment priority. Eur J Orthod.

[16] Daniels C, Richmond S (2000). The development of the index of complexity, outcome and need (ICON). J Orthod.

[17] Linder-Aronson S (1974) Orthodontics in the Swedish public dental health service. Trans Eur Orthod Soc:233–240 4534974

[18] Bundesamt für Sozialversicherung (BSV), Schweizerische Zahnärzte-Gesellschaft (SSO) (2009) Informationen für Zahnarzt und Zahnärzte über die Eidgenossische Invaliditatsversicherung. URL: https://www.sso.ch/fileadmin/upload_sso/2_Zahnaerzte/1_Informationen/Zaz-Infos_BSV_IV_SSO_Nov_2010_D_2_.pdf. Accessed:10–06–2020

[19] Gesch D, Kirbschus A, Schröder W, Bernhardt O, Proff P, Bayerlein T, Gedrange T, Kocher T (2006). Influence of examiner differences on KIG-classification when assessing malocclusions. J Orofac Orthop.

